# A versatile papaya mosaic virus (PapMV) vaccine platform based on sortase-mediated antigen coupling

**DOI:** 10.1186/s12951-017-0289-y

**Published:** 2017-07-18

**Authors:** Ariane Thérien, Mikaël Bédard, Damien Carignan, Gervais Rioux, Louis Gauthier-Landry, Marie-Ève Laliberté-Gagné, Marilène Bolduc, Pierre Savard, Denis Leclerc

**Affiliations:** 1 Department of Microbiology, Infectiology and Immunology, Infectious Disease Research Center, Laval University, 2705 Boul. Laurier, Quebec City, PQ G1V 4G2 Canada; 2 Neurosciences, Laval University, 2705 Boul. Laurier, Québec City, PQ G1V 4G2 Canada

**Keywords:** Papaya mosaic virus, Flexuous rod shape nanoparticles, Vaccine platform, Influenza M2e based vaccine, Sortase, Transpeptidase

## Abstract

**Background:**

Flexuous rod-shaped nanoparticles made of the coat protein (CP) of papaya mosaic virus (PapMV) have been shown to trigger innate immunity through engagement of toll-like receptor 7 (TLR7). PapMV nanoparticles can also serve as a vaccine platform as they can increase the immune response to fused peptide antigens. Although this approach shows great potential, fusion of antigens directly to the CP open reading frame (ORF) is challenging because the fused peptides can alter the structure of the CP and its capacity to self assemble into nanoparticles—a property essential for triggering an efficient immune response to the peptide. This represents a serious limitation to the utility of this approach as fusion of small peptides only is tolerated.

**Results:**

We have developed a novel approach in which peptides are fused directly to pre-formed PapMV nanoparticles. This approach is based on the use of a bacterial transpeptidase (sortase A; SrtA) that can attach the peptide directly to the nanoparticle. An engineered PapMV CP harbouring the SrtA recognition motif allows efficient coupling. To refine our engineering, and to predict the efficacy of coupling with SrtA, we modeled the PapMV structure based on the known structure of PapMV CP and on recent reports revealing the structure of two closely related potexviruses: pepino mosaic virus (PepMV) and bamboo mosaic virus (BaMV). We show that SrtA can allow the attachment of long peptides [Influenza M2e peptide (26 amino acids) and the HIV-1 T20 peptide (39 amino acids)] to PapMV nanoparticles. Consistent with our PapMV structural model, we show that around 30% of PapMV CP subunits in each nanoparticle can be fused to the peptide antigen. As predicted, engineered nanoparticles were capable of inducing a strong antibody response to the fused antigen. Finally, in a challenge study with influenza virus, we show that mice vaccinated with PapMV-M2e are protected from infection.

**Conclusions:**

This technology will allow the development of vaccines harbouring long peptides containing several B and/or T cell epitopes that can contribute to a broad and robust protection from infection. The design can be fast, versatile and can be adapted to the development of vaccines for many infectious diseases as well as cancer vaccines.

**Electronic supplementary material:**

The online version of this article (doi:10.1186/s12951-017-0289-y) contains supplementary material, which is available to authorized users.

## Background

The use of nanoparticles as vaccine platforms is a very promising approach to improve immune response directed against poorly immunogenic antigens. Nanoparticles can be made of viral structural proteins that self-assemble to mimic the native organisation and conformation of a viral pathogen [1–9]. They can also present peptide antigens on their surface and induce a potent humoral response [2–4, 10]. Being of a size similar to viruses, nanoparticles are efficiently phagocyted and processed by antigen presentation cells (APCs) such as dendritic cells (DC) [11, 12]. They can also activate innate immunity through pathogen associated molecular patterns (PAMPs), which are recognized by innate receptors (pathogen recognition receptors; PRRs) [13, 14]. Therefore, the use of nanoparticles to display heterologous epitopes is a promising strategy for the development of novel vaccines.

We have developed a novel rod-shaped viral nanoparticle made of the coat protein (CP) of papaya mosaic virus (PapMV) that is self-assembled around a ssRNA. PapMV nanoparticles trigger innate immunity efficiently [14] through engagement of toll-like receptor 7 (TLR7) [15]. After phagocytosis, nanoparticles reach the endosome of immune cells and liberate the ssRNA, which engages and activates TLR7 [15]. PapMV nanoparticles can be used as an adjuvant for improvement of vaccines [16], an immune enhancer for the treatment of cancer in immunotherapy [17], or as a vaccine platform to trigger an immune response to a specific peptide antigen [18]. Fusion of peptides directly to the open reading frame (ORF) of the PapMV CP leads to the formation of chimeric nanoparticles that can trigger either a humoral [19, 20] or a CTL response against the fused antigen [21–24]. This approach has shown the great potential of PapMV nanoparticles as a vaccine platform, but has also revealed the challenges involved in their engineering. Indeed, the fusion of peptides to the CP ORF can alter its structure and affect its capacity to self-assemble into immunogenic nanoparticles. We showed previously that the fusion of long peptides interferes with self-assembly [20].

To decrease this stress on the structure of the nanoparticles, we propose here a novel method for coupling of peptides to already self-assembled nanoparticles using the bacterial transpeptidase, sortase A (SrtA). SrtA anchors surface proteins, such as virulence factors, to the bacterial cell wall peptidoglycans of Gram-positive bacteria [25–27]. Target proteins become linked to a polyG motif at the N-terminus of the acceptor peptidoglycan through the recognition and cleavage of their LPXTG recognition motif by SrtA. SrtA has been used widely for the site-specific labelling of proteins with a wide range of functional groups, such as fluorescent labels and green fluorescent protein (GFP) [28–31], biotin [32], PEG [29, 31, 33], peptide nucleic acids [34], lipids [35], sugars [36], and other proteins [30, 37, 38]. A soluble form of SrtA lacking the transmembrane domain can be produced efficiently in *E. coli* and used in in vitro transpeptidation reactions.

Based on in silico modelling of the 3D structure of full length PapMV CP and of PapMV, we engineered the PapMV vaccine platform with the receptor motif of SrtA, and used SrtA to attach long peptides to the nanoparticles. This technology allows rapid and efficient coupling of peptide antigens to PapMV nanoparticles without affecting their structure. PapMV nanoparticles with SrtA-conjugated influenza M2e peptide were shown to be immunogenic, and induced protection against influenza infection.

## Results

### Structural models of PapMV CP and PapMV

**Fig. 1:**
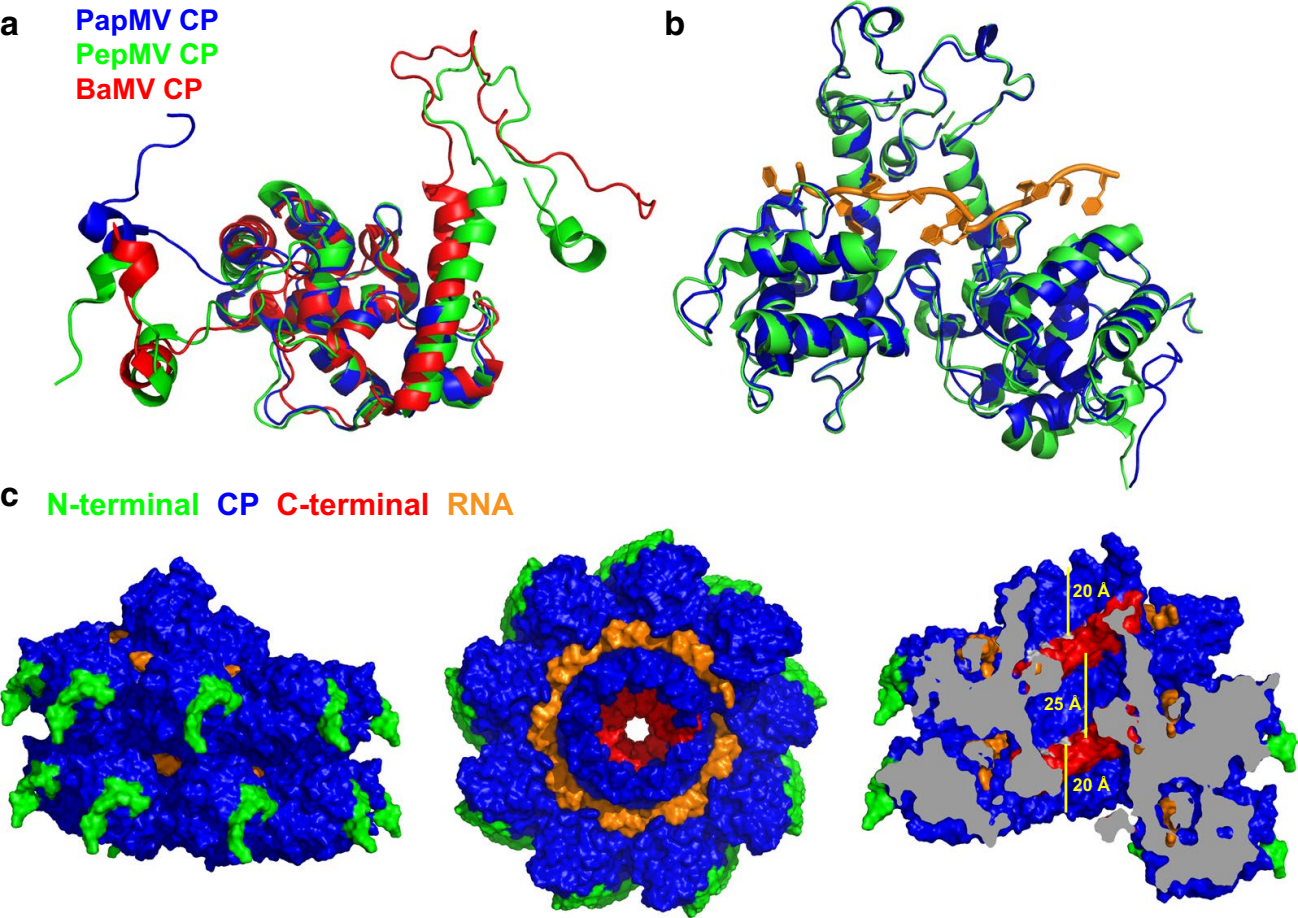
Modelling of the PapMV CP and PapMV structure. **a** The full-length structure of the PapMV CP was modelled based on the recently published structure of two members of the potexvirus group: BaMV CP and PepMV CP. The core region of PapMV CP (PDB 4DOX, *blue*) superimposes well on the core region of PepMV CP (PDB 5FN1, *green*) and BaMV CP (PDB 5A2T, *red*). **b** Superposition of two subunits of the PapMV model (*blue*) with two CP subunits of the PepMV structure (*green*, PDB 5FN1) demonstrates the concordance between the two structures. **c** To show how each of the PapMV CP interacts with each other, and with the ssRNA in the nanoparticle, we modeled the self assembly of the 18 subunits (~2 turns) that comprise PapMV nanoparticles. The CP N-terminal residues (10 first) are shown in *green*, the CP core in *blue*, the CP C-terminus in *red* (10 last residues), and the RNA is in *orange*. The last C-terminal residue of the CP is displayed in *light grey* in the cutaway view on the *right*, and the *bars* represent 20 Å—the distance separating CP C-terminal residues from the PapMV nanoparticle exterior at both extremities, and 32 Å—the distance between two CP C-terminal residues separated by one capsid turn

The recent solving of the near-atomic structure of Bamboo mosaic virus (BaMV) [39] and Pepino mosaic virus (PepMV) [40] by cryo-electron microscopy (cryo-EM), has allowed the structural details of *Potexvirus* viruses to be revealed at an atomic level. Interestingly, the CPs of these two *Potexviruses* share high structural conservation with the structure of the truncated CP of PapMV (PDB ID 4DOX) [41] with root mean square deviations (RMSD) of around 1.6 and 3 Å for the backbone heavy atoms of PepMV (PDB ID 5FN1) and BaMV (PDB ID 52AT), respectively (Fig. 1a). Moreover, previous low resolution cryo-EM data reported by our group on PapMV [41] demonstrates that it adopts a capsid symmetry similar to that adopted by BaMV and PapMV, consisting of a left-handed helix of ~130Å in diameter, with ~10 CP subunits per turn and a pitch of ~35 Å. These structural similarities prompted us to elaborate a structural model for PapMV based on BaMV and PapMV to guide the development of our nanoparticles following a rational design approach. Briefly, a homology model of the complete PapMV CP based on the structure of the truncated PapMV CP (PDB ID 4DOX), BaMV CP and PepMV CP was generated using I-TASSER [42]. The complete PapMV CP was then aligned on the subunit CPs of PepMV (PDB 5FN1) to construct the model (Fig. 1b). The viral ssRNA (in orange) was positioned by homology with PepMV (Fig. 1b, c).

As observed for the structures of BaMV and PepMV, the N-terminal ends of PapMV CPs are exposed on the surface of the virus, while the C-terminii are located in the central cavity (Fig. 1c). Therefore, fusions to the N-terminus can modify the surface of PapMV nanoparticles and affect the interface of interaction between nanoparticles and immune cells. Fusions made at the C-terminus could cause steric hindrance in the central cavity, interfering with nanoparticle assembly, as indicated by the cross-section view of the PapMV nanoparticle model displayed in Fig. 1c (right illustration) and as previously reported [20]. Considering that the C-terminal sections of two CPs separated by one capsid turn define a cavity of around 25 Å (Fig. 1c), it is anticipated that the longest C-terminal fusion allowed in the core of the nanoparticles would be ~17 amino acids if arranged as an α-helix. But, fusion of long peptides on each side of the nanoparticles where the C-terminus are accessible is possible without changing the surface of the nanoparticle. Therefore, it suggests that the development of a method allowing peptide coupling to available C-terminus at the extremity of already-assembled nanoparticles is an attractive approach for the coupling of long peptides to our vaccine platform.

### Engineering of the PapMV nanoparticles with the SrtA recognition motif

**Fig. 2:**
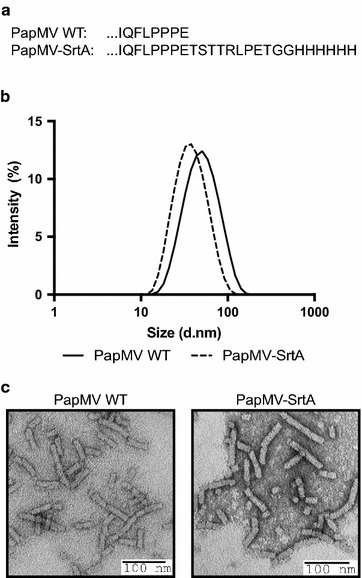
Engineering of PapMV coat protein carrying the SrtA recognition motif. **a** Amino acid sequence of the PapMV CP C-terminus of the WT CP as compared to the engineered PapMV CP that harbours a linker of 5 amino acids (TSTTR) followed by the SrtA recongnition motif (LPETGG); a 6×His tag was inserted at the C-terminus of the PapMV coat protein (named PapMV-SrtA). **b** The length of the rod-shaped nanoparticles was assessed by dynamic light scattering (DLS). PapMV-SrtA (40 nm) was showed to be slightly smaller than the WT PapMV (54 nm). **c** Transmission electron microscopy (TEM) of WT PapMV (*left*) and PapMV-SrtA (*right*) nanoparticles shows the rod shape of the nanoparticles

Based on the structural model of the PapMV nanoparticle presented in Fig. 1c, we chose to fuse the sortase A (SrtA) LPETGG recognition motif followed by a 6×H tag to the C-terminus of the PapMV CP. This fusion is preceded by the linker TSTTR, which was introduced to allow the LPETGG motif to reach the exterior of the nanoparticles at both ends (Fig. 1c-right panel), making it available for the transpeptidation reaction. Indeed, assuming a maximal length of 3.5 Å per amino acid, the PapMV structural model suggests that around 5 amino acids are required to span the 20 Å distance separating CP C-terminal residues from the nanoparticle exterior at both extremities (Fig. 1c-right panel). Fusion of the receptor motif did not alter the capacity of the CP to self-assemble around the ssRNA, leading to generation of nanoparticles (PapMV-SrtA) with an average length of 40 nm vs 54 nm for the WT PapMV nanoparticle, as shown by dynamic light scattering (DLS) (Fig. 2a). The width of the DLS curve in the two types of nanoparticles was comparable, suggesting that nanoparticles are of different lengths ranging from ~20 to ~100 nm. Electron microscopy (EM) confirmed that PapMV-WT and PapMV-SrtA are similar in appearance (Fig. 2b). As a control, we engineered another PapMV CP lacking the TSTTR linker preceding the SrtA recognition motif [PapMV-SrtA (short)] (Additional file 1: Figure S1). This construct is predicted to not permit coupling because the SrtA recognition motif is hidden too far within the core cavity of the PapMV nanoparticle. As with PapMV-SrtA, the fusion did not interfere with self-assembly, and generated nanoparticles comparable to those of WT PapMV, as confirmed by DLS (Additional file 1: Figure S1B) and electron microscopy (Additional file 1: Figure S1C).

### SrtA-mediated coupling of peptides onto PapMV nanoparticles

To achieve SrtA-mediated coupling onto PapMV nanoparticles, we chose two long peptides of 26 amino acids (influenza M2e peptide) and 39 amino acids (HIV-1 T20 peptide), respectively. The peptide M2e is derived from the extracellular domain of the matrix protein 2 (M2e) of influenza virus. M2e is highly conserved in most influenza A strains [43] and is a valuable antigen in inducing protection to influenza infection [19, 44]. The T20 peptide is derived from the surface glycoprotein gp41 of human immunodeficiency virus (HIV-1) [45]. A monoclonal antibody (2F5) directed towards this peptide was shown to neutralise HIV-1 infection in vitro, and has been proposed for use as a vaccine antigen [46].

**Fig. 3:**
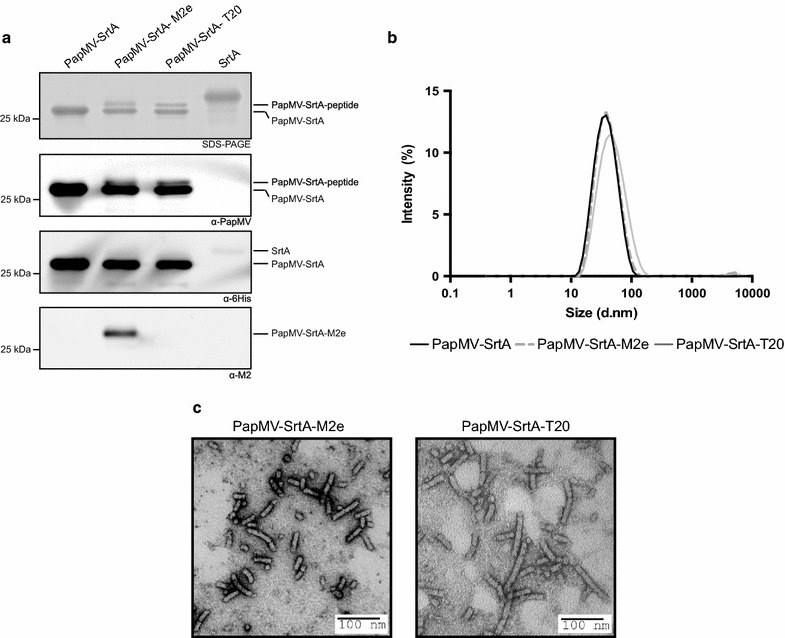
Coupling of T20 and M2e onto PapMV-SrtA nanoparticles. **a** To reveal the efficacy of coupling of peptides onto the PapMV vaccine platform, we performed a 5% Tris–Glycine SDS-PAGE. The coupling induced by the sortase is covalent, and induced a shift on the gel as compared with the WT PapMV CP. Western blot of SrtA-conjugated nanoparticles PapMV-SrtA-M2e and PapMV-SrtA-T20 revealed fusion of the peptide on the PapMV CP. Western blots were directed against the PapMV CP, the 6×H tag, or the M2e peptide, as indicated under each* panel*. To evaluate if coupling of the peptide affected the structure of the nanoparticles, we evaluated their size by DLS analysis (**b**), comparing PapMV-SrtA (54 nm) to conjugated PapMV-SrtA-M2e (43 nm) and PapMV-SrtA-T20 (52 nm) nanoparticles. We also used TEM of PapMV-SrtA-M2e (*left*) and PapMV-SrtA-T20 (*right*) nanoparticles to confirm that the rod shape was preserved after the coupling experiment

We performed transpeptidation reactions using 25 µM of PapMV-SrtA nanoparticles (based on the amount of PapMV CP evaluated by BCA) incubated with 50 µM of either the M2e or T20 peptide and 50 µM of SrtA. Following the coupling reaction, the SrtA and the free peptides were removed by filtration, and the samples were loaded on SDS-PAGE to reveal the coupling efficiency (Fig. 3a-top panel). PapMV CP coupled to either the M2e or the T20 peptide migrated as slightly higher molecular weight proteins than the uncoupled PapMV-SrtA CP on SDS-PAGE (Fig. 3a). The identity of the higher band was further confirmed by immunoblotting using specific antibodies directed towards PapMV CP (Fig. 3a-second panel), or the M2e peptide (Fig. 3a-fourth panel). Also, we used a specific antibody directed to the 6×H tag that, as expected, reacted only with the uncoupled PapMV-SrtA CP (Fig. 3a-third panel). To assess the coupling efficacy precisely, we calculated the ratio between the intensity of the coupled CP and that of the total CP (coupled + uncoupled) on SDS-PAGE (Fig. 3a), which revealed a coupling efficacy of 35% with the M2e peptide and 32% with the T20 peptide. We performed six coupling reactions and always obtained a similar coupling efficiency, which ranged from 26 to 38% for M2e and 30 to 34% for the T20 peptide (not shown). To evaluate whether the structure of the nanoparticles was affected by the coupling reaction, we compared the length of the PapMV-SrtA nanoparticles with those conjugated with M2e or T20 peptides (Fig. 3b). The nanoparticles were similar in length compared to PapMV-SrtA, as shown by DLS (Fig. 3b), and in their appearance in EM (Fig. 3c).

As expected from our prediction based on the structure of the PapMV-SrtA CP, only a fraction (about 1/3) of the CP reacted with SrtA. We observed that coupling was saturated with 50 µM of peptide and no additional coupling was observed when 100 or 200 µM was used (Additional file 2: Figure S2). Finally, we confirmed that coupling reactions with the PapMV-SrtA(short) (Additional file 3: Figure S3A) were very inefficient as compared to those with PapMV-SrtA, which shows the importance of the linker (TSTTR) in making the SrtA recognition motif solvent-accessible (Additional file 3: Figure S3B). Coupling on PapMV-SrtA(short) nanoparticles was consistently ineffective (four repeated experiments), unlike coupling on the PapMV-SrtA construct as revealed by immunoblot using an antibody directed against the M2e peptide (Additional file 3: Figure S3B).

### The PapMV-SrtA vaccine platform enhances the humoral response to the T20 peptide

**Fig. 4:**
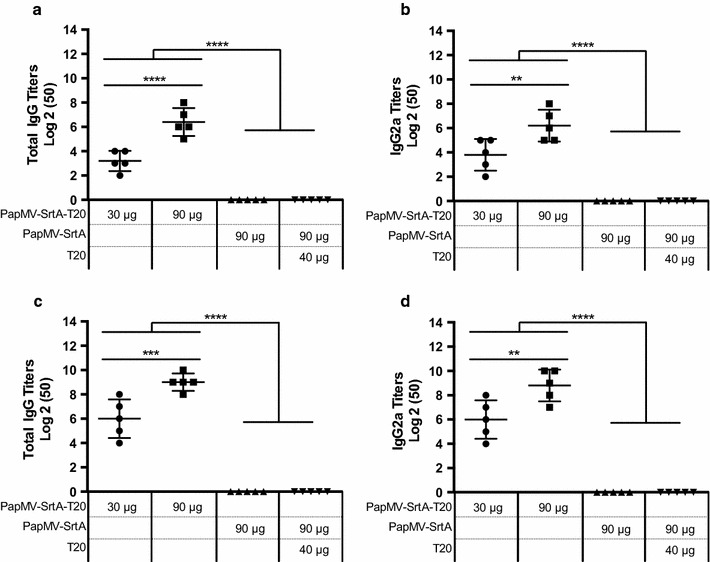
PapMV-SrtA-T20 induces a T20 specific antibody response. We immunized animals [Female Balb/C mice (5/group)] twice, with a 14-day interval by the intramuscular route with either 30 or 90 µg of PapMV-SrtA-T20, PapMV-SrtA or T20 peptide, to evaluate if coupling of T20 peptides fused to the surface of PapMV nanoparticles enhance the humoral response to the T20 peptide antigen. Blood was taken 14 days following each immunization, and ELISA assays were performed to evaluate levels of anti-T20 total IgG (**a**) or IgG2a (**b**) after the first immunization, and anti-T20 total IgG (**c**) or IgG2A (**d**) after the booster immunization. ****P < 0.0001 all PapMV-SrtA-T20 groups against PapMV-SrtA alone, or PapMV-SrtA and T20 peptide. ***P < 0.001 between 30 and 90 µg of PapMV-SrtA-T20 total IgG (d27), ****P < 0.0001 total IgG (d13) and **P < 0.01 IgG2a (d13 and d27)

To assess the capacity of the PapMV-SrtA vaccine platform to improve the humoral response to the T20 peptide, we immunized Balb/C mice (5/group) twice, with a 14-day interval, with 90 µg of PapMV-SrtA, 30 or 90 µg of PapMV-SrtA-T20, or 90 µg of PapMV-SrtA with 40 µg of free T20 peptide. Blood levels of total anti-T20 IgG and IgG2a were assessed by ELISA 13 days after each immunization (Fig. 4a–d). Mice vaccinated with PapMV-SrtA or PapMV-SrtA and the free T20 peptide did not develop any detectable T20 immunoglobulins. However, 30 µg of PapMV-SrtA-T20 was sufficient to mount a robust antibody response against the T20 peptide after a single immunization (Fig. 4ab). This result confirms that coupling of the peptide to the PapMV vaccine platform improved the immunogenicity of the peptide significantly. We also observed a rapid class switch towards a TH1 response, since a strong IgG2a response specific to the T20 peptide was recorded after the first immunization (Fig. 4b), which is expected with the PapMV vaccine platform as previously reported [19, 44, 47, 48]. The IgG2a isotype is known to show a higher avidity for the antigen, and is therefore more efficient for neutralization of a viral infection. Finally, and as expected, the boost immunization raised the total IgG and IgG2a titers significantly (Fig. 4c, d).

### The PapMV vaccine platform enhances the humoral response to the M2e peptide and induces protection to an influenza challenge

**Fig. 5:**
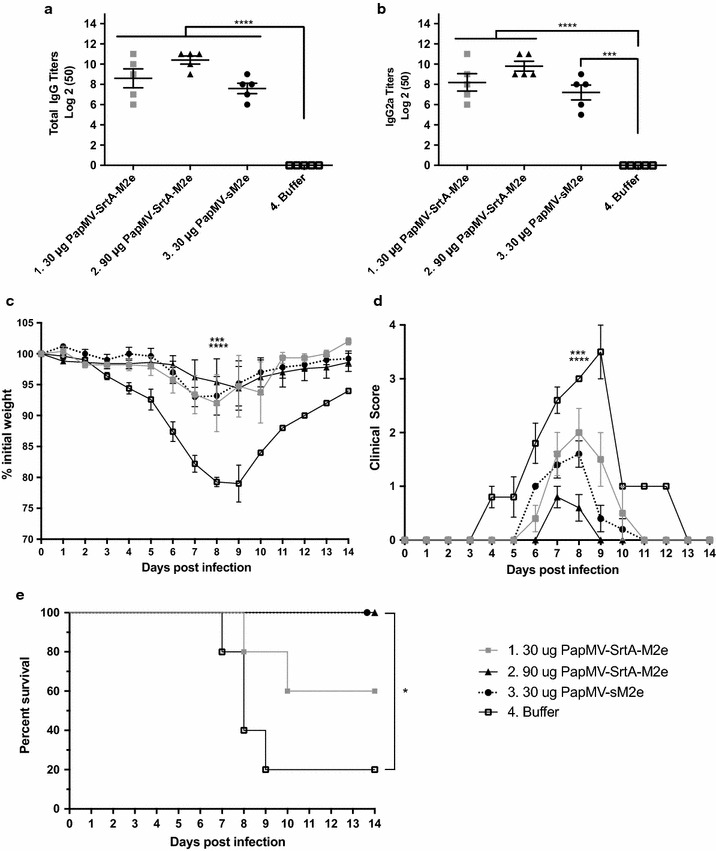
PapMV-SrtA-M2e induces a specific anti-M2e immune response and protection against an influenza challenge. The M2e peptide has been previously shown to be a highly conserved epitope of influenza A virus that is able to induce protection against an influenza challenge. Therefore, Female Balb/C mice (5 per group) were immunized twice with PapMV nanoparticles coupled to the M2e peptide using the sortase (PapMV-SrtA-M2e), PapMV-sM2e [where a small version of the M2e (sM2e) is fused directly to the N-terminus of the PapMV CP] (positive control), or buffer of the vaccine formulation alone. Blood was taken 13 days following the last immunization, and ELISA assays performed to evaluate levels of anti-M2e total IgG (**a**) or IgG2a (**b**). **P < 0.01 for groups 2 vs 3 total IgG titers and * P< 0.05 for group 2 vs 3 IgG2a titers. To assess the capacity of the PapMV-SrtA-M2e nanoparticles to induce protection to an influenza challenge, immunized mice were infected with 1 × LD80 of influenza A/WSN/33 virus 14 days after the last immunization, and followed for clinical symptoms and survival for 14 days. **c** Mean clinical score of infection signs on a scale of 0 to 4. ***P < 0.001 for group 1 vs 4, and for group 2 vs 3, ****P < 0.0001 for groups 2–3 vs 4, and for group 1 vs 2, all at day 8 post-challenge. **d** Mean weight loss expressed as percentage of initial weight. ***P < 0.001 for group 1 vs 4, and ****P < 0.0001 for group 2–3 vs 4. **e** Survival of mice expressed as Kaplan–Meier survival curves. *P < 0.5 for groups 2–3 vs 4

To evaluate the efficacy of our approach in an animal infectious model, we immunized mice with PapMV-SrtA-M2e, and evaluated the humoral response directed towards the M2e peptide, and the capacity to provide protection to an influenza challenge. We also included in this study, as a reference, the PapMV-sM2e construct previously generated in our laboratory [19]. PapMV-sM2e harbours a fusion of a 9-amino-acid long peptide (EVETPIRNE) corresponding to the central region of the M2e peptide. In these nanoparticles, every PapMV CP molecule harbours a fusion of the small M2e peptide. This construct was previously shown to induce production of antibodies to the full length M2e peptide, and to provide protection to an influenza challenge [19]. To compare the efficacy of the two constructs, five mice per group received prime and booster immunizations at intervals of 14 days with either 30 or 90 µg of PapMV-SrtA-M2e, 30 µg PapMVCP-sM2e or formulation buffer. Thirteen days following the first and the second immunization, sera were collected, and levels of total IgG and IgG2a directed against the M2e peptide were measured by ELISA. Interestingly, the levels of antibodies directed against the M2e (total IgG and IgG2a) were similar between all groups following the first immunization (Additional file 4: Figure S4). After two immunizations, total IgG and IgG2a were comparable between groups immunized with 30 µg of PapMV-SrtA-M2e or 30 µg of PapMV-sM2e (Fig. 5a, b). However, immunization with 90 µg of PapMV-SrtA-M2e triggered a humoral response against the M2e peptide that was significantly higher than that of 30 µg of either PapMV-SrtA-M2e or PapMV-sM2e (Fig. 5a).

To evaluate the capacity of the candidate vaccine to protect against influenza, we challenged immunized mice 14 days after the second immunization with 1×LD80 of influenza A/WSN/33 virus. This experiment was critical because it allowed us to demonstrate that the nanoparticle vaccine platform not only improves the humoral response to a peptide antigen, but also induces production of antibodies that protect the animal against an influenza infection. After the challenge, three different assessments were used to confirm the efficacy of the vaccine formulation: (1) weight loss, which gives a reliable readout because infected mice loose weight rapidly following an influenza challenge. Because weight can be evaluated precisely each day following the challenge, it becomes an important readout to evaluate the efficacy of the protection; (2) assessment of symptoms, which is an empirical measurement that usually correlates with weight loss. Symptoms were assessed by technicians blind to treatment group to decrease the risk of assessment bias; (3) survival, which is the ultimate proof of protection induced by a vaccine formulation. To comply with the animal ethics protocol of our institution, we sacrificed animals that lost more than 20% of their body weight.

In brief, mice immunized with PapMV-SrtA-M2e (30 and 90 µg) and PapMV-sM2e (30 µg) showed minimal weight loss as compared to mice immunized with formulation buffer (Fig. 5c). Clinical signs of infection (ruffled fur, curved back, mobility loss) were significantly reduced in mice immunized with 90 µg of PapMV-SrtA-M2e, although weight loss was not significantly different between mice immunized with PapMV-SrtA-M2e or PapMV-sM2e (Fig. 5c, d). Formulation buffer immunized mice showed severe clinical symptoms and significant weight loss, reaching a mean of 79.3% of their initial weight, at day 8 post-infection. Only groups immunized with 90 µg of PapMV-SrtA-M2e or with PapMV-sM2e showed 100% survival. While 60% of mice immunized with 30 µg of PapMV-SrtA-M2e survived the infection, only 20% of formulation buffer immunized mice survived (Fig. 5e). To confirm this result, we repeated the same experiment with 10 mice per group, evaluated the humoral response after two immunizations (Additional file 5: Figure S5AB), and performed the challenge with 1LD80 of the WSN/33 influenza strain. We monitored weight loss (Additional file 5: Figure S5C), symptoms (Additional file 5: Figure S5D) and survival (Additional file 5: Figure S5E). Consistent with the previous experiment (Fig. 5), the two best performing vaccine formulations were PapMV-sM2e and PapMV-SrtA-M2e, which induced a significant protection to influenza challenge.

## Discussion

In this study, we built a 3D model of PapMV CP and PapMV nanoparticles based on the recently published structures of two other members of the potexvirus family: BaMV [39] and PepMV [40]. This model was used to refine engineering of the PapMV vaccine platform. We confirmed experimentally that a fusion of a 17 amino acid peptide harbouring the SrtA recognition motif to the C-terminus of the CP was tolerated, and did not interfere with self-assembly. As anticipated from our PapMV structure prediction, only a fraction of CP subunits in the nanoparticle seem to present the SrtA recognition motif to the sortase, leading to coupling efficiencies that reached a plateau at around 30% for both peptides tested. Coupling probably occurs on the extremities of the nanoparticles, leaving the surface free for interactions with immune cells. In fact, even if the reaction was pushed by increasing the amount of peptide, the amount of coupling did not increase, suggesting that we had reached saturation of all available sites. According to the structural model, the C-termini of the CP units are located in the interior cavity of the nanoparticle, and are thus not available for the coupling reaction. The coupling of peptide antigens to already pre-assembled nanoparticles allowed attachment of longer peptides (26 and 39 amino acids) without affecting the structure of the nanoparticle. Consistent with our model, fusion of a 24-amino-acid peptide to the CP C-terminus led to the formation of unstable nanoparticles that were less immunogenic [20]. With the SrtA approach, we can fuse longer peptides containing multiple epitopes—a major advantage when developing new vaccines.

While SrtA has been widely used for the modification and conjugation of diverse proteins including antibodies, protein–protein, and protein-to-solid-support conjugates, few studies have investigated the modification of nanoparticles by SrtA-mediated conjugation. A recent study showed that SrtA could be used to link FITC or GFP to the N-terminus of cowpea chlorotic mottle virus (CCMV) coat protein expressing an N-terminal glycine residue [28]. Although the authors of the latter study did not use SrtA to functionalize the coat proteins post-assembly, they were able to co-assemble conjugated and unconjugated CCMV into VLPs to encapsulate the cargo. In another study, M13 bacteriophage surface proteins were modified using SrtA from *Staphylococcus aureus* and *Streptococcus pyogenes* [49]. Various proteins and small molecules have been attached to either the pIII, pIX or pVIII bacteriophage proteins expressing N-terminal glycines but the potential of this approach as a vaccine platform has not been tested through immunization of animals. Both these studies used SrtA to modify the N-terminus of the CP, while, in contrast, our system conjugates peptides to the C-terminus of the CP to minimise any modification of the nanoparticle surface that may interfere with the interaction with immune cells. Finally, fusion of the SrtA recognition motif to an internal surface-exposed loop of the hepatitis B virus (HBV) core protein led to efficient conjugation of a peptide derived from the enterovirus 71 (EV71) and the AD-4 domain on glycoprotein B (gB) of human cytomegalovirus (HCMV) on HBV icosahedral nanoparticles [50]. These SrtA-modified HBV nanoparticles improved the immune responses directed towards their antigen.

PapMV nanoparticles engineered with the SrtA recognition motif were shown to significantly improve the humoral response triggered towards the attached peptides. Although only one-third of the CPs in PapMV-SrtA nanoparticles became coupled to the M2e or T20 peptide, a robust immune response was triggered and PapMV-SrtA-M2e was capable of protecting mice from an influenza challenge. The main differences between the PapMV vaccine platform and the HBV nanoparticle are the shape of the particle and their differential ability to trigger innate immunity: HBV is icosahedral whereas PapMV is rod-shaped. Furthermore, PapMV has the unique capacity to trigger innate immunity through the engagement of TLR7, which results in the induction of IFN-α secretion [15, 17]—a major advantage for the development of an efficient vaccine. The PapMV vaccine platform is also capable of inducing a class switch toward production of IgG2a antibodies, which implies cooperation of CD4+ immune cells [51]. IgG2a antibodies control viral infection better than other immunoglobulins due to their higher avidity and their capacity to trigger the antibody-dependent cell-mediated toxicity (ADCC) that is critical to eliminate virus-infected cells with natural killer (NK) cells [52].

Considering the speed at which a candidate vaccine can be developed, and the versatility of this approach, we believe that our SrtA method is advantageous for the presentation of antigens on the PapMV vaccine platform. Sometimes an antigen alone is sufficient to provide protection but, in most cases, this is a very limiting factor.

## Conclusion

The covalent attachment of antigens onto PapMV nanoparticles using SrtA allows fusion of long peptides (26–39 amino acids) to a vaccine platform. The covalent linkage of the antigen to the platform enhanced significantly the immune response directed to the antigens, and provided protection to challenge with a viral infection (influenza). This approach is more versatile than fusion of the antigen directly to the ORF of the PapMV CP—a route that tolerates fusion of only small peptides. Fusion of longer peptides to the ORF can impair CP structure and its capacity to self-assemble into nanoparticles, and formation of nanoparticles is essential to obtain a strong immune response. Thus, the SrtA-based tagging of peptide antigens onto already pre-formed PapMV nanoparticles allows for fast, flexible and easy nanoparticle modification, representing a promising tool in vaccine design against infectious diseases, as well as in the development of vaccines in cancer immunotherapy or tailor-made vaccine candidates for personalised medicine [17].

## Methods

### Production and purification of PapMV nanoparticles

The PapMV nanoparticles harbouring the C-terminal LPETGG SrtA recognition site with (PapMV-SrtA) or without (PapMV-StrA9short) the TSTTR linker, and PapMV-M2e and PapMV nanoparticles were kindly provided by Folia Biotech (Quebec City, Quebec, Canada). The nanoparticles were produced and purified as described elsewhere [19]. Briefly, PapMV CPs were produced in *E. coli*. Purified CPs were assembled in vitro with a non-coding synthetic ssRNA derived from the native sequence of papaya mosaic virus RNA. Levels of contaminant LPS were measured by a Limulus Amebocyte Lysate assay (LAL) (Lonza, Walkersville, Maryland, USA). The LPS concentration in the final nanoparticle samples was always below 50 endotoxin units (EU)/mg of protein, and was thus considered negligible. The amount of nanoparticles used to perform each experiment was estimated using the BCA method (Thermo ScientificTM PierceTM BCA Protein Assay), a detergent-compatible formulation based on bicinchoninic acid (BCA) for the colorimetric detection and quantitation of total protein. Since PapMV CP is the main component of the nanoparticles (>95% of the total nanoparticle weight), it is a reliable method to quantify the amount of nanoparticles used in the experiments conducted in this study.

### Expression and purification of recombinant sortase A

Expression and purification of recombinant sortase A followed the method described in [53], with the following modifications: (1) 2×YT (16 g bacto-tryptone, 10 g yeast extract, 5 g NaCl, pH 7.0) culture medium was used instead of LB medium, (2) IPTG induction was carried out when culture OD_600nm_ reached 1.0 ± 0.1, (3) *E. coli* was harvested by one 15-min centrifugation at 9000×*g* at 4 °C, (4) bacterial pellet from 1 L of culture was suspended in 40 mL of cold lysis buffer (50 mM Tris–HCl pH 7.5, 500 mM NaCl, 10 mM imidazole), (5) bacteria were lysed by three passages through an Emulsiflex C5 homogenizer (Avestin, Ottawa, Ontario, Canada) at 15,000–25,000 psi, (6) 100 U of benzonase (Sigma-Aldrich Inc. Saint-Louis, Missouri, USA) and 5 mM of MgCl_2_ were added to the cell lysate and incubated for 20 min with gentle agitation before cell lysate clarification by centrifugation at 20,442×*g* for 20 min, (7) the clarified supernatant was applied to a 40 mL IMAC column packed with Ni Sepharose 6 Fast Flow Resin (GE Healthcare Life Sciences, Little Chalfont, Buckinghamshire, United Kingdom), and washed with 10 column volumes (CV) of lysis buffer and 5 CV of wash 2 buffer (50 mM Tris–HCl pH 7.5, 150 mM NaCl, 25 mM imidazole). Purification on the IMAC column was done with a ÄKTA purifier 10 FPLC (GE Healthcare Life Sciences). Imidazole was removed using a Sartoflow(R) Slice 200 benchtop crossflow system for diafiltration with a 10 kDa cutoff (Sartorius, Göttingen, Germany). LPS contaminants were removed on an ion exchange membrane bound to quaternary ammonium ligands using the ÄKTA purifier 10, and measured by the LAL assay (Lonza, Basel, Switzerland). LPS levels were under 50 EU/mg of protein and were considered negligible. The yield of SrtA was approximately 100 mg/L of culture.

### Peptides

Synthetic peptides, with over 90% purity, used for SrtA labelling, GGG-M2e (GGGSLLTEVETPIRNEWGCRCNDSSD) and GGG-T20 (GGGYTSLIHSLIEESQNQQ EKNEQELLELDKWASLWNWF) were purchased from GenScript (United States) and Biomatik (Canada), respectively.

### Evaluation of the structural properties of nanoparticles by dynamic light scattering and transmission electron microscopy

Nanoparticle size was determined by dynamic light scattering (DLS) with a ZetaSizer Nano ZS (Malvern, Malvern, Worcestershire, United Kingdom). PapMV nanoparticles were diluted to 0.12 mg/mL in 10 mM Tris–HCl, pH 8.0, and loaded into a disposable plastic cuvette. Samples were measured four times, and average results generated by cumulative analysis.

Nanoparticle shape was assessed by transmission electron microscopy (TEM). Samples were diluted to 0.02 mg/mL and mixed at a 1:1 ratio with a 3% solution of uranyl acetate for 7 min in the dark; 8 µL of this mixture was then loaded on carbon formvar grids for 5 min in the dark. Excess solution was absorbed and grids were air-dried for at least 2 h before observation. Grids were observed with a FEI-TECNAI-Spirit transmission electron microscope (FEI, Hillsboro, Oregon, USA). DLS and TEM were carried out on the nanoparticles before and after the sortase A transpeptidation reaction to assess the effect of the ligation on the physical properties of the nanoparticles.

### Sortase A—mediated transpeptidation reaction

SrtA conjugation reactions were performed in 1X SrtA reaction buffer (50 mM Tris–HCl pH 8.0, 150 mM NaCl, 10 mM CaCl_2_). Reactions containing 50 µM of SrtA, 50–200 µM of GGG-peptide and 25 µM of different PapMV constructions bearing the SrtA recognition motif LPETGG in nanoparticles were incubated for 2.5 h at room temperature. The molar concentration of nanoparticles was calculated based on the protein concentration of the CP as determined by BCA assay, knowing that 1 kDa = 1 g/mole and 1 µM = 0.000001 mol/L. Reactions were stopped with the addition of EGTA to a final concentration of 10 µM. For the PapMV-SrtA-M2e- and PapMV-SrtA-T20-conjugated samples used in the animal studies, unconjugated peptides and SrtA were removed with an amicon centrifugal filter unit with a cutoff size of 100 kDa (EMD Millipore, Darmstadt, Germany). PapMV-SrtA nanoparticles, being larger than 100 kDa, were retained by the filter unit. Conjugation efficiency was analyzed by 10% Tris-Tricine or 15% Tris–Glycine SDS-PAGE colored with Sypro-Ruby gel stain (Life Technologies Inc. Carlsbad, California, USA). We used 15% Tris–Glycine SDS-PAGE gels for analysis of the conjugation reaction for better resolution of the PapMV-SrtA and PapMV-SrtA coupled to the peptide (Fig. 3; Additional file 2: Figure S2). SrtA reactions seen in Fig. 3 and Additional file 2: Figure S2 were directly analyzed on SDS-PAGE without eliminating the SrtA enzyme excess peptide by amicon 100 kDa.

### SDS-PAGE analysis for peptide coupling quantification

Peptide-labelled PapMV-SrtA nanoparticles were diluted to 0.1 µg/µL in cathode buffer for Tris-Tricine gels, or in Tris–Glycine migration buffer supplemented with 30% of SDS loading buffer (50% glycerol, 2% SDS, 0.002% bromophenol blue, 14% 2-mercaptoethanol). Samples were then heated at 95 °C for 10 min and 4 µL of solution (0.4 µg) was loaded on 10% Tris-Tricine SDS-PAGE for the gels shown in Fig. 4 or 15% Tris/Glycine SDS-Page for the gels shown in Fig. 3 and Additional file 2: Figure S2. Gels were colored with Sypro-Ruby gel stain (Life Technologies Inc. Carlsbad, California, USA) following the manufacturer’s rapid staining protocol. Sypro-Ruby fluorescence was detected with the 610 nm emission filter following excitation using the green laser (532 nm) of the Typhoon 9200 imager (GE Healthcare Life Sciences). The fluorescence signal resulting from protein bands was quantified using the image analysis software ImageQuant 5.2, and the intensity volumes associated to the different bands, corrected for background signal, were used for the conjugation quantification analysis. The conjugation efficiency was calculated by dividing the signal of coupled PapMV-SrtA protein band by the sum of conjugated and unconjugated PapMV-SrtA signals.

### Western blot

After SDS-PAGE migration, proteins were transferred onto PVDF Immobilon^®^-FL membranes (EMD Millipore, Darmstadt, Germany) and blocked using bløck™-FL buffer (EMD Millipore). Membranes were incubated overnight with the following primary antibodies diluted in the bløck™-FL buffer: rabbit anti-PapMV polyclonal antibody, mouse anti-influenza A M2 (14C2) monoclonal antibody (Santa Cruz Biotechnology Inc. Dallas, Texas, USA) or mouse anti-His_6_ polyclonal antibody (Roche, Basel, Switzerland). After washing with PBS/0.1% Tween-20, membranes were incubated for 2 h with either Alexa Fluor^®^ 532 coupled goat anti-rabbit IgG (H+L) (Life Technologies Inc. Carlsbad, California, USA) or Alexa Fluor^®^ 488 coupled goat anti-mouse IgG (H+L) (Life Technologies Inc.). After washing, membranes were dried to reduce background, and fluorescent signals were captured using the Typhoon 9200 imager (GE Healthcare Life Sciences).

### Animals and immunization

Six- to 8-week-old female BALB/c mice were immunized by intramuscular injection (i.m.) twice, 2 weeks apart, with buffer (50 µL of 10 mM Tris–HCl, pH 8.0) or PapMV-SrtA-M2e, PapMV-sM2e, PapMV-SrtA-T20 or PapMV-SrtA with free T20 peptide. Blood samples were collected before each immunization on days 13 and 27. Serum was separated from the blood by centrifugation in BD Microtainer SST blood collection tubes (BD, East Rutherford, New Jersey, USA) for 2 min at 10,000×*g*. Total IgG and IgG2a serotype endpoint titers against M2e and T20 peptides in the sera of immunized mice were determined by enzyme linked immunosorbent assay (ELISA) as described below.

### Enzyme linked immunosorbent assay

M2e (CSLLTEVETPIRNEWGCRCNDSSD) and T20 (GGGYTSLIHSLIEESQNQQEK NEQELLELDKWASLWNWF) peptides were coated overnight at 4 °C in 96-well flat bottom nunc™ MaxiSorp plates (VWR, Radnor, Pennsylvania, USA) at 1 and 2.5 µg/mL, respectively, in 100 µL 0.1 M NaHCO_3_ buffer, pH 9.6. Plates were blocked with 150 µL per well of PBS/0.1% Tween-20/2% BSA, and washed three times with PBS/0.1% Tween-20, before adding mice sera in twofold serial dilutions starting at 1:50. Plates were incubated for 1.5 h at 37 °C, and washed four times in PBS/0.1% Tween-20 before adding the peroxidase-conjugated goat anti-mouse IgG or IgG2a secondary antibody (Jackson ImmunoResearch Laboratories, West Grove, Pennsylvania, USA). After washing the plates four times, peptide-specific antibodies were detected with the addition of TMB substrate (Fitzgerald Industries International, Acton, Massachusetts, USA). The reaction was stopped with the addition of 0.18 M H_2_SO_4_. Results are expressed as antibody endpoint titers greater than threefold the OD_450nm_ of the background value of the pre-immune sera at the same dilution.

### Influenza challenge

Two weeks following the last immunization, immunized mice were infected with 1 LD80 of mouse-adapted influenza A/WSN/33 (H1N1). Mice were lightly anesthetized with isoflurane, and infected with 50 µL of virus by intranasal instillation. Weight loss and clinical signs of disease were monitored for 14 days post-infection. Symptoms were rated from 0 to 4 as follows: (0) no symptoms: (1) lightly spiked fur and curved back; (2) spiked fur and curved back; (3) difficulty moving and slight dehydration; and (4) severe dehydration, lack of reflexes and ocular secretions. Mice reaching a score of 4, or having lost 20% or more of their initial body weight, were euthanized.

### Statistical analysis

Differences (antibody titers, weight and symptoms) between immunized groups were measured by Holm-Šídák multiple comparisons test or Tukey’s multiple comparisons test. Survival differences were evaluated by Kaplan–Meier survival analysis. *P*-values <0.05 were considered statistically significant. Statistical analyses were performed using Graph-Pad Prism version 6.0 (GraphPad Software, La Jolla California USA, http://www.graphpad.com).

## Additional files

**Additional file 1: Figure S1.** Engineering of PapMV coat protein carrying the SrtA recognition motif missing the linker. (A) The SrtA recognition motif (LPETGG) of SrtA was inserted into the C-terminus of the PapMV coat protein (CP). (B) The size of the VLPs was assessed by dynamic light scattering (DLS). PapMV-SrtA(short) (65 nm) was showed to be slightly longer than the WT PapMV (54 nm). (C) Transmission electron microscopy (TEM) of WT PapMV (left) and PapMV-SrtA(short) (right) nanoparticles.

**Additional file 2: Figure S2.** Effect of peptide concentrations on the SrtA coupling reaction. (A) Comparison of the C-terminal sequence of PapMV WT, PapMV-SrtA and PapMV-SrtA(short). In PapMV-SrtA, a linker of 5 amino acids (TSTTR) was added before the SrtA recognition motif LPETGG, while in PapMV-SrtA(short) the recongnition motif was included directly in the native PapMV sequence by deleting two proline residues. (B) SDS-PAGE and western blot of SrtA reactions on PapMV-SrtA and PapMV-SrtA(short) nanoparticles. PapMV nanoparticles (25 µM) were incubated with SrtA (50 µM) and GGG-M2e peptide (50 µM) for 2.5 hours at room temperature. Reactions were stopped with EGTA (10 µM) and passed through a 100 kDa centrifugal filter unit to eliminate excess peptide and contaminating SrtA. PapMV nanoparticles retained by the 100 kDa filter unit were diluted to 0.1 µg/µL in migration buffer supplemented with 30% of SDS loading buffer and 4 µL was loaded onto 15% Tris-Glycine SDS-PAGE. PapMV-SrtA, PapMV-SrtA(short) and SrtA controls correspond to lanes 1, 10 and 11, respectively. Lanes 2-5 and lanes 6-9 represent four experimental replicates of SrtA conjugation on PapMV-SrtA or PapMV-SrtA(short), respectively. Efficient SrtA labelling of GGG-M2e peptide onto PapMV nanoparticles was assessed by SDS-PAGE (top panel), and immunoblotting with a specific antibody against the M2 (bottom panel).

**Additional file 3: Figure S3.** Optimization of the coupling reaction. SDS-PAGE and Western Blots of SrtA reactions in the presence of PapMV-SrtA nanoparticules and increasing concentrations of GGG-M2e peptide. Western blots were directed against the PapMV CP, the 6xH tag, or the M2e peptide, as indicated on the bottom of each panel. Target products are shown by a red dash. SrtA reactions were diluted to obtain a PapMV-SrtA concentration of 0.1µg/µL (based on molar concentration in the reaction) in migration buffer supplemented with 30% of SDS loading buffer, and 4 µL was loaded onto 10% Tris-Tricine SDS-PAGE for analysis.

**Additional file 4: Figure S4.** PapMV-SrtA-M2e induces a specific anti-M2e immune response after a single immunization. Female Balb/C mice, 5 per group, were immunized twice with the indicated formulations. Mice were bled 13 days after the first immunization, and levels of anti-M2e total IgG (A) and IgG2a (B) were measured by ELISA. ****P<0.0001 for groups 1, 2, 3 vs 4 for total IgG and IgG2a titers.

**Additional file 5: Figure S5.** PapMV-SrtA-M2e induces a specific anti-M2e immune response and protection against an influenza challenge-(repeat). Female Balb/C mice (10 per group) were immunized twice with 30 µg PapMV-SrtA-M2e, 90 µg PapMV-SrtA-M2e, 30 µg PapMV-sM2e or formulation buffer. At 13 days following the last immunization, mice were bled and ELISA assays performed to evaluate levels of anti-M2e total IgG (A) or IgG2a (B). ****P<0.0001 for groups 1, 2, 3 vs 4 total IgG titers and IgG2a titers. Mice were infected with 1 x LD80 of influenza A/WSN/33 virus 14 days after the last immunization, and followed for clinical symptoms and survival for 14 days. (C) Mean weight loss expressed as percentage of initial weight. **P<0.01 for group 1 vs 2, ***P<0.001 for group 3 vs 4, and for group 2 vs 3, ****P<0.0001 for group 2 vs 4, all at day 7 post-challenge. (D) Mean clinical score of infection signs on a scale of 0 to 4. ***P<0.001 for group 1 vs 3, and ****P<0.0001 for group 1 vs 2 and groups 2, 3 vs 4. (E) Survival of mice expressed as Kaplan-Meier survival curves. *P<0.5 for groups 2 vs 4 and **P<0.01 for group 3 vs 4.
