# Supplementary figures and images for: A versatile papaya mosaic virus (PapMV) vaccine platform based on sortase-mediated antigen coupling
Source: J Nanobiotechnology. 2017 Jul 18;15:54. doi: 10.1186/s12951-017-0289-y (PMC5516373; doi:10.1186/s12951-017-0289-y)

A

PapMV WT: ...IQFLPPPE  
 PapMV-SrtA(short): ...IQFLP----ETGGHHHHHH

B

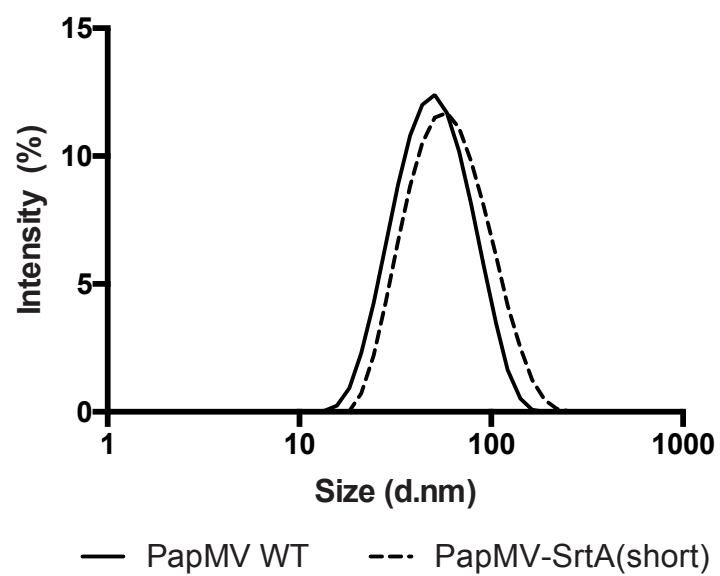

C

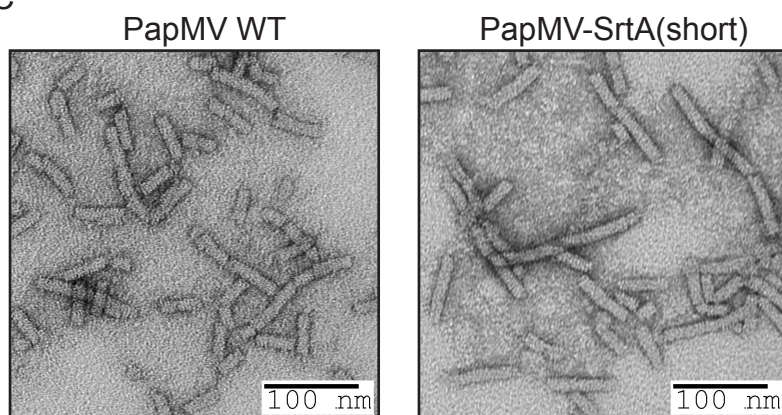

Supplement: Supplementary file 1 — Additional file 1: Figure S1. Engineering of PapMV coat protein carrying the SrtA recognition motif missing the linker. (A) The SrtA recognition motif (LPETGG) of SrtA was inserted into the C-terminus of the PapMV coat protein (CP). (B) The size of the VLPs was assessed by dynamic light scattering (DLS). PapMV-SrtA(short) (65 nm) was showed to be slightly longer than the WT PapMV (54 nm). (C) Transmission electron microscopy (TEM) of WT PapMV (left) and PapMV-SrtA(short) (right) nanoparticles. [file 12951_2017_289_MOESM1_ESM.pdf]

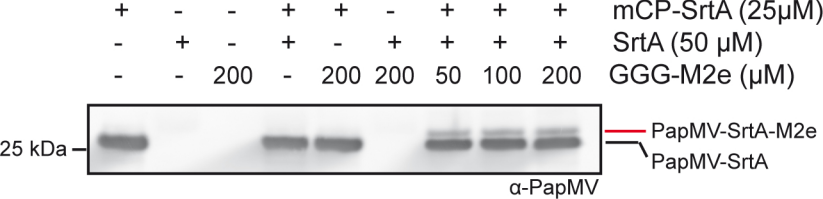

Supplement: Supplementary file 2 — Additional file 2: Figure S2. Effect of peptide concentrations on the SrtA coupling reaction. (A) Comparison of the C-terminal sequence of PapMV WT, PapMV-SrtA and PapMV-SrtA(short). In PapMV-SrtA, a linker of 5 amino acids (TSTTR) was added before the SrtA recognition motif LPETGG, while in PapMV-SrtA(short) the recongnition motif was included directly in the native PapMV sequence by deleting two proline residues. (B) SDS-PAGE and western blot of SrtA reactions on PapMV-SrtA and PapMV-SrtA(short) nanoparticles. PapMV nanoparticles (25 µM) were incubated with SrtA (50 µM) and GGG-M2e peptide (50 µM) for 2.5 hours at room temperature. Reactions were stopped with EGTA (10 µM) and passed through a 100 kDa centrifugal filter unit to eliminate excess peptide and contaminating SrtA. PapMV nanoparticles retained by the 100 kDa filter unit were diluted to 0.1 µg/µL in migration buffer supplemented with 30% of SDS loading buffer and 4 µL was loaded onto 15% Tris-Glycine SDS-PAGE. PapMV-SrtA, PapMV-SrtA(short) and SrtA controls correspond to lanes 1, 10 and 11, respectively. Lanes 2-5 and lanes 6-9 represent four experimental replicates of SrtA conjugation on PapMV-SrtA or PapMV-SrtA(short), respectively. Efficient SrtA labelling of GGG-M2e peptide onto PapMV nanoparticles was assessed by SDS-PAGE (top panel), and immunoblotting with a specific antibody against the M2 (bottom panel). [file 12951_2017_289_MOESM2_ESM.pdf]

A

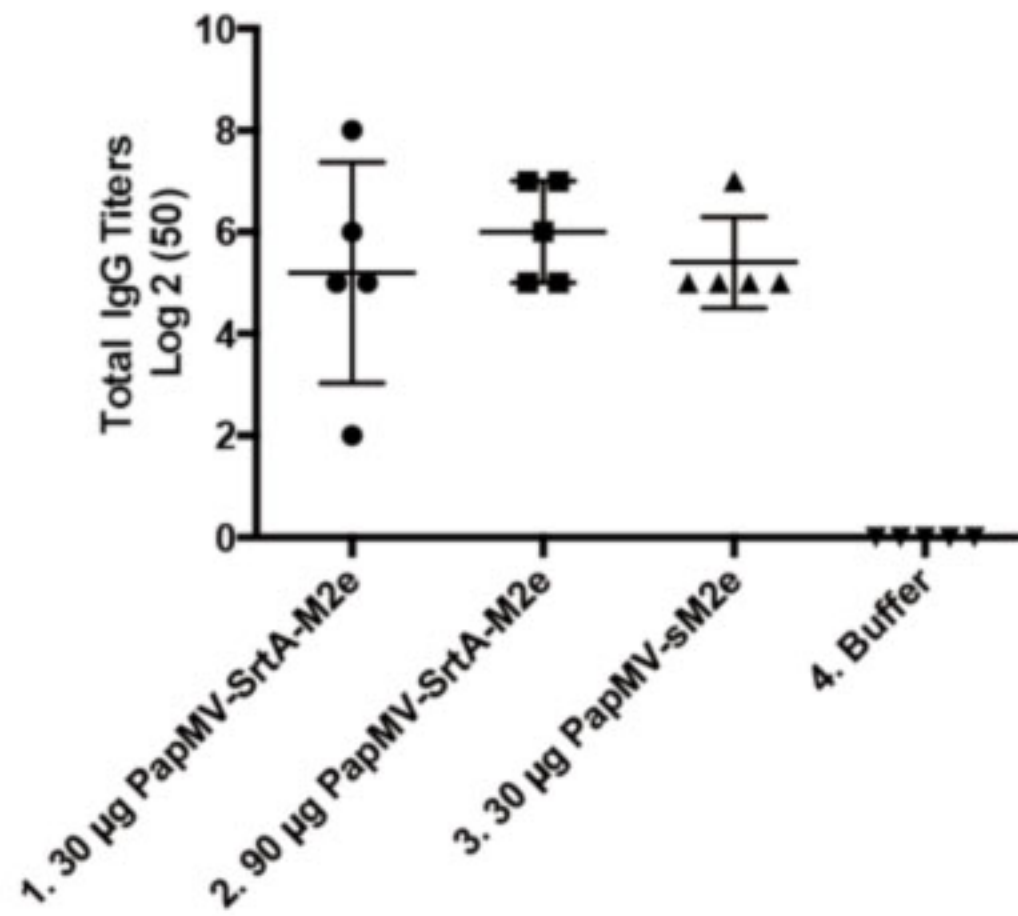

B

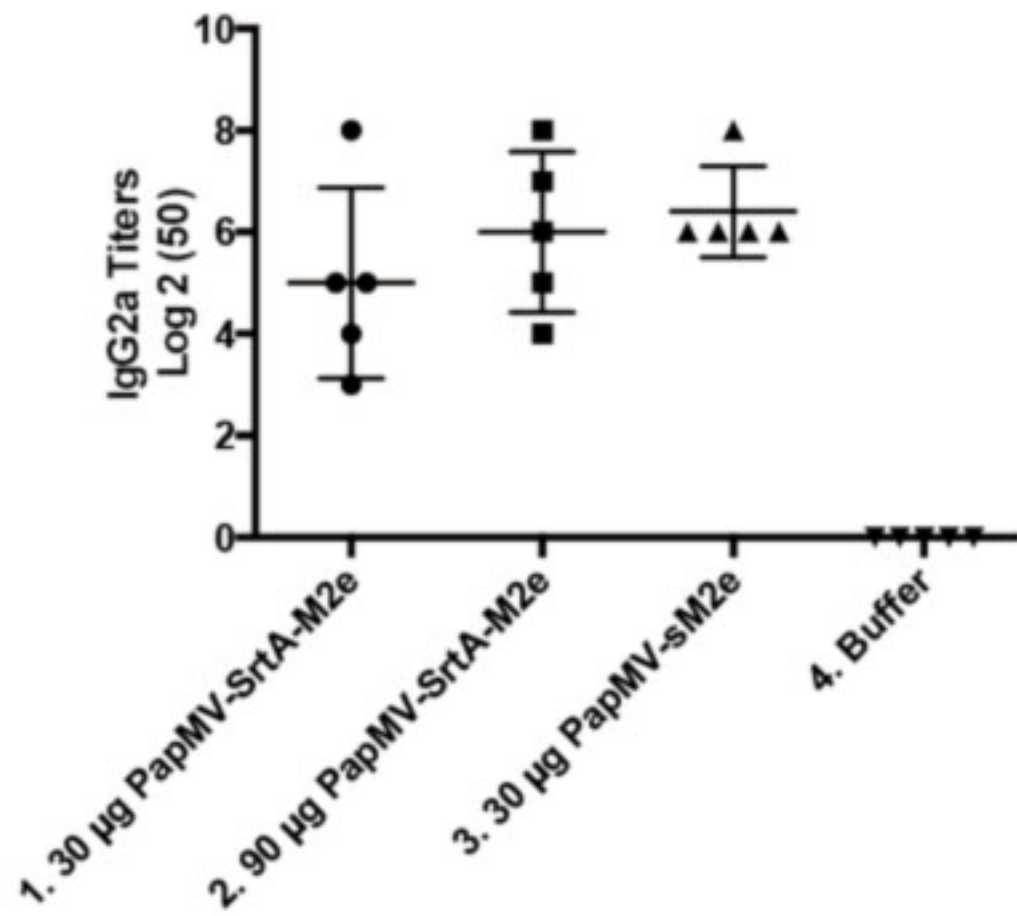

Supplement: Supplementary file 4 — Additional file 4: Figure S4. PapMV-SrtA-M2e induces a specific anti-M2e immune response after a single immunization. Female Balb/C mice, 5 per group, were immunized twice with the indicated formulations. Mice were bled 13 days after the first immunization, and levels of anti-M2e total IgG (A) and IgG2a (B) were measured by ELISA. ****P<0.0001 for groups 1, 2, 3 vs 4 for total IgG and IgG2a titers. [file 12951_2017_289_MOESM4_ESM.pdf]

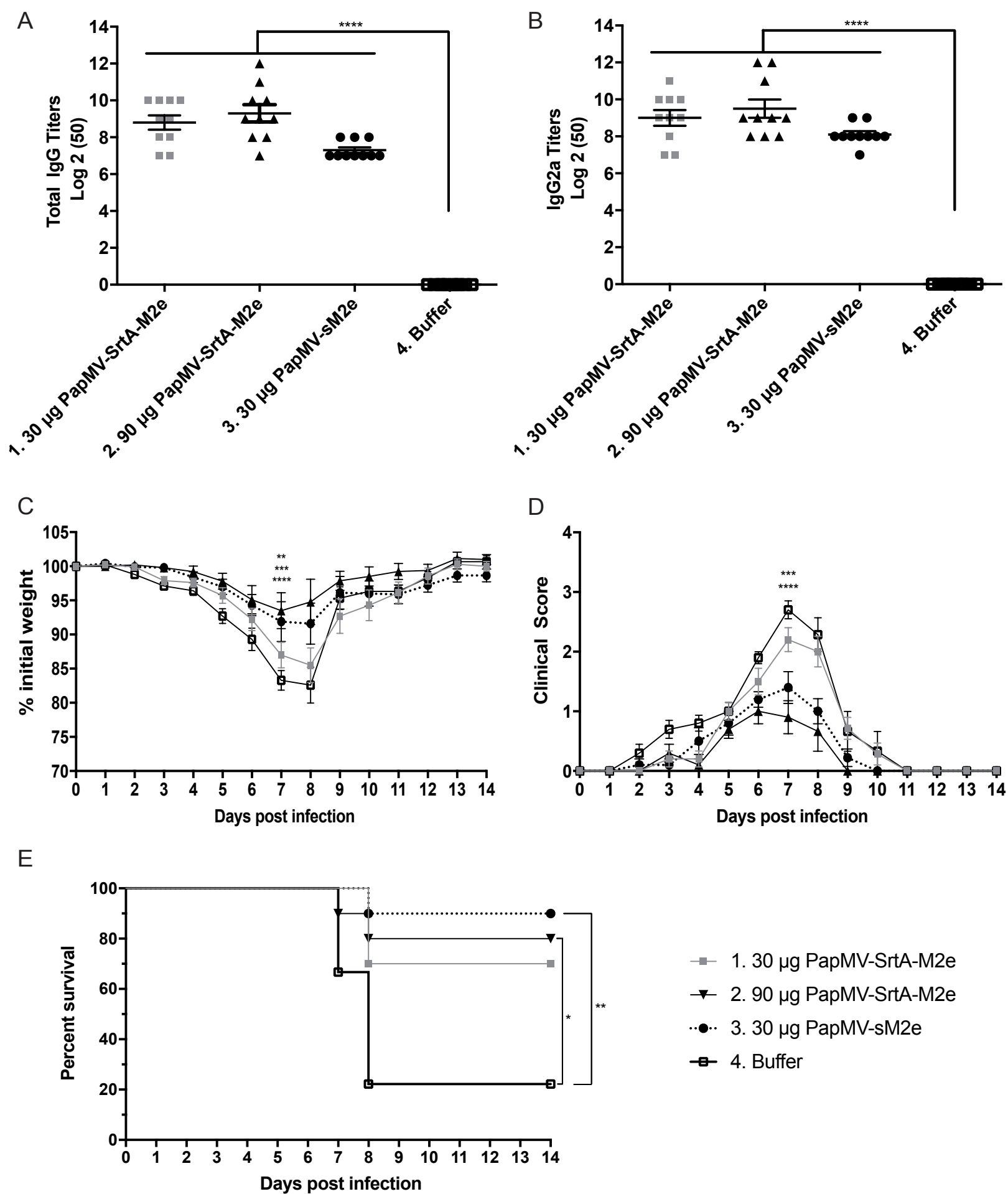

Supplement: Supplementary file 5 — Additional file 5: Figure S5. PapMV-SrtA-M2e induces a specific anti-M2e immune response and protection against an influenza challenge-(repeat). Female Balb/C mice (10 per group) were immunized twice with 30 µg PapMV-SrtA-M2e, 90 µg PapMV-SrtA-M2e, 30 µg PapMV-sM2e or formulation buffer. At 13 days following the last immunization, mice were bled and ELISA assays performed to evaluate levels of anti-M2e total IgG (A) or IgG2a (B). ****P<0.0001 for groups 1, 2, 3 vs 4 total IgG titers and IgG2a titers. Mice were infected with 1 x LD80 of influenza A/WSN/33 virus 14 days after the last immunization, and followed for clinical symptoms and survival for 14 days. (C) Mean weight loss expressed as percentage of initial weight. **P<0.01 for group 1 vs 2, ***P<0.001 for group 3 vs 4, and for group 2 vs 3, ****P<0.0001 for group 2 vs 4, all at day 7 post-challenge. (D) Mean clinical score of infection signs on a scale of 0 to 4. ***P<0.001 for group 1 vs 3, and ****P<0.0001 for group 1 vs 2 and groups 2, 3 vs 4. (E) Survival of mice expressed as Kaplan-Meier survival curves. *P<0.5 for groups 2 vs 4 and **P<0.01 for group 3 vs 4. [file 12951_2017_289_MOESM5_ESM.pdf]
