# Supplementary material for: A versatile papaya mosaic virus (PapMV) vaccine platform based on sortase-mediated antigen coupling
Source: J Nanobiotechnology. 2017 Jul 18;15:54. doi: 10.1186/s12951-017-0289-y (PMC5516373; doi:10.1186/s12951-017-0289-y)

A

PapMV WT: ...IQFLPPPE
PapMV-SrtA: ...IQFLPPPETSTTRLPETGGHHHHHH
PapMV-SrtA(short): ...IQFLP----ETGGHHHHHH

B

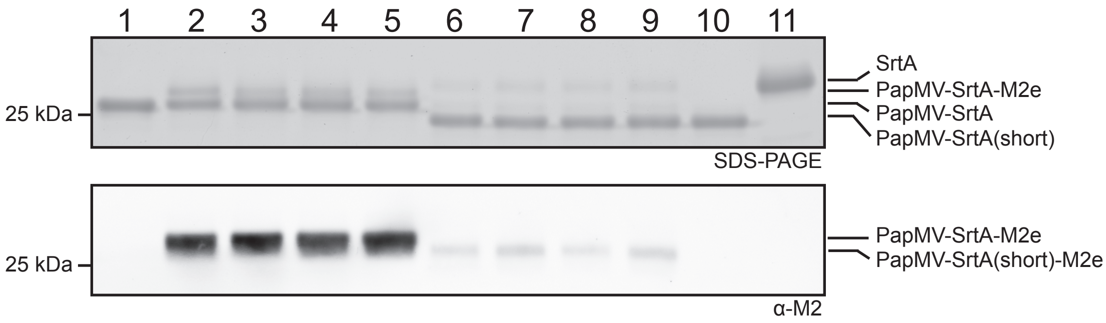

Supplement: Supplementary file 3 — Additional file 3: Figure S3. Optimization of the coupling reaction. SDS-PAGE and Western Blots of SrtA reactions in the presence of PapMV-SrtA nanoparticules and increasing concentrations of GGG-M2e peptide. Western blots were directed against the PapMV CP, the 6xH tag, or the M2e peptide, as indicated on the bottom of each panel. Target products are shown by a red dash. SrtA reactions were diluted to obtain a PapMV-SrtA concentration of 0.1µg/µL (based on molar concentration in the reaction) in migration buffer supplemented with 30% of SDS loading buffer, and 4 µL was loaded onto 10% Tris-Tricine SDS-PAGE for analysis. [file 12951_2017_289_MOESM3_ESM.pdf]
